# Over-expression of AtPAP2 in *Camelina sativa *leads to faster plant growth and higher seed yield

**DOI:** 10.1186/1754-6834-5-19

**Published:** 2012-04-02

**Authors:** Youjun Zhang, Laura Yu, Ka-Fu Yung, Dennis YC Leung, Feng Sun, Boon L Lim

**Affiliations:** 1 School of Biological Sciences, the University of Hong Kong, Pokfulam, Hong Kong, China; 2 Department of Mechanical Engineering, the University of Hong Kong, Pokfulam, Hong Kong, China; 3 Department of Applied Biology and Chemical Technology, The Hong Kong Polytechnic University, Hung Hom, Kowloon, China

**Keywords:** AtPAP2, *Camelina sativa*, Fatty acid, Photosynthesis, SPS, Sucrose

## Abstract

**Background:**

Lipids extracted from seeds of *Camelina sativa *have been successfully used as a reliable source of aviation biofuels. This biofuel is environmentally friendly because the drought resistance, frost tolerance and low fertilizer requirement of *Camelina sativa *allow it to grow on marginal lands. Improving the species growth and seed yield by genetic engineering is therefore a target for the biofuels industry. In *Arabidopsis*, overexpression of purple acid phosphatase 2 encoded by *Arabidopsis *(*AtPAP2*) promotes plant growth by modulating carbon metabolism. Overexpression lines bolt earlier and produce 50% more seeds per plant than wild type. In this study, we explored the effects of overexpressing AtPAP2 in *Camelina sativa*.

**Results:**

Under controlled environmental conditions, overexpression of AtPAP2 in *Camelina sativa *resulted in longer hypocotyls, earlier flowering, faster growth rate, higher photosynthetic rate and stomatal conductance, increased seed yield and seed size in comparison with the wild-type line and null-lines. Similar to transgenic *Arabidopsis*, activity of sucrose phosphate synthase in leaves of transgenic *Camelina *was also significantly up-regulated. Sucrose produced in photosynthetic tissues supplies the building blocks for cellulose, starch and lipids for growth and fuel for anabolic metabolism. Changes in carbon flow and sink/source activities in transgenic lines may affect floral, architectural, and reproductive traits of plants.

**Conclusions:**

Lipids extracted from the seeds of *Camelina sativa *have been used as a major constituent of aviation biofuels. The improved growth rate and seed yield of transgenic *Camelina *under controlled environmental conditions have the potential to boost oil yield on an area basis in field conditions and thus make *Camelina*-based biofuels more environmentally friendly and economically attractive.

## Background

Crop plants obtain energy entirely by photosynthesis; this autotrophic mechanism has long been a target of research aiming to maximize crop production [1]. Sugars derived from photosynthesis are energy sources for metabolism and are building blocks of complex carbohydrates [2]. Sugars also regulate expression of photosynthetic genes in mesophyll and interact with other signaling pathways involved in plant growth regulation [3]. Previous studies suggest that photosynthetic performance can be improved by modifying sugar-signaling mechanisms [3]. A large number of protein kinases, protein phosphatases and transcription factors are involved in these pathways [4]. Constitutive expression of *Arabidopsis *purple acid phosphatase 2 (AtPAP2) by the CaMV35s promoter in *Arabidopsis *enhances plant growth and seed yield by modulating carbon metabolism [5]. Both sucrose phosphate synthase (SPS) activity and sucrose content are elevated in leaves of transgenic lines. Overexpressed lines grow faster and produce > 50% more seeds per plant than wild type [5]. AtPAP2 is targeted to both chloroplasts and mitochondria by its C-terminal hydrophobic motif; removal of this motif eliminates growth-promoting phenotypes, indicating that its subcellular localization within the two key energy-generating organelles in plants is important for its biological functions [5]. Purple acid phosphatases (PAPs) constitute a family of acidic binuclear metalloenzymes that hydrolyse phosphate esters and anhydrides under acidic conditions [6]. Plant PAPs are generally considered to mediate phosphorus acquisition and redistribution through their ability to hydrolyze phosphorus compounds [7,8]. Some PAPs have biological functions other than those involved in P metabolism. For example, certain PAPs, such as GmPAP3 [9] and AtPAP17 [10], have peroxidase activity. The tobacco purple acid phosphatase NtPAP12 dephosphorylates phosphoryl residues of apoplasticproteins [11]. The enzyme is actively transcribed in the process of cell wall regeneration [12]. AtPAP2 is currently the only PAP known to be involved in carbon metabolism.

*Arabidopsis thaliana *and *Camelina sativa *are members of the Angiosperm Family *Brassicaceae *[13]. *Camelina sativa*, or false flax, has been cultivated for oil in Europe, Central Asia and North America. Compared with rapeseed, the cultivation of *Camelina *has become vanishingly small since World War II [14]. Currently, *Camelina *is being grown to produce aviation biofuels. To date, Japan Airline, Air New Zealand and the US Navy (F/A18) have successfully completed test flights with a 50/50 blend of *Camelina*-based biofuel and petroleum-based jet fuel. With its low requirements for water and fertilizers, *Camelina *could be grown on marginal land as a low-input biofuel crop [15]. With an oil content of > 40% polyunsaturated fatty acids (linoleic acid, 18:2n-6 and α-linolenic acid, 18:3n-3), of which, linolenic acid makes up ~35%, *Camelina *seed is also an important source of omega-3 fatty acids [16].

Through overexpression of AtPAP2, transgenic *Camelina *lines grow faster and produce more seeds than wild-type (WT) plants under controlled environmental conditions. These phenotypes are attributable to elevated photosynthetic rates and SPS activity in leaves. Higher seed yield would make cultivation of *Camelina *more economical and environmentally-friendly. High seed yield per unit area would enhance biofuel production and reduce consumption of fossil fuels, thus reducing CO_2_, methane (CH_4_) and nitrous oxide (N_2_O) emissions.

## Results

### AtPAP2 overexpression accelerated *Camelina *growth

**Figure 1:**
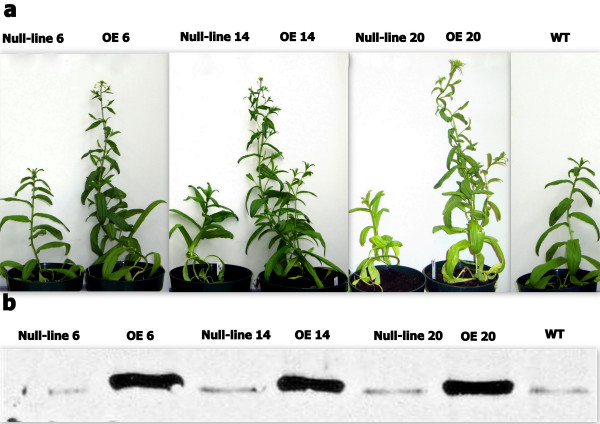
**Growth phenotype of transgenic *Camelina *at day 40**. a, All three OE lines produced more branches, grew faster, and flowered earlier than the WT and null-lines. b, Expression of AtPAP2 in OE lines was confirmed by Western blotting using AtPAP2-specific antibodies. OE lines had higher expression of AtPAP2, while there was a homologous protein of AtPAP2 in *Camelina*.

Multiple independent overexpression lines of *Camelina sativa *with AtPAP2 protein expression were obtained through *Agrobacterium* transformation. After confirmation by genomic PCR and western blotting (Figure 1), three independent T2 AtPAP2 overexpression (OE) lines were selected for characterization. When there was a 3:1 segregation of T_1 _transgenic seeds, null-lines were selected for their relatively short hypocotyl lengths on MS plates. Identities of the three null-lines were confirmed by genomic PCR and western blotting (Figure 1). A weak western blot signal was observed in protein extracts from the null lines and WT. We attribute this signal to likely cross-reactivity between the AtPAP2 antibody and the endogenous AtPAP2-like protein (91% amino acid sequence identity) that has been detected in transcriptome analysis of *Camelina sativa *leaves (unpublished data). All plants were grown on MS agar for a week and then transferred to a growth chamber (150 μmol photons/m^2^/s) under a 16 h light 22°C/8 h dark 18°C photoperiod/temperature regimen. The plants were transferred immediately after first flowering to greenhouse conditions at 22°C under Hong Kong's natural day/night photoperiod. Compared to WT and their respective null-lines, all three AtPAP2 overexpressor lines (OE6, OE14 and OE20) grew faster and produced more lateral branches (Figure 1). Typically, plants of the three OE lines started flowering 7-9 days earlier than WT and null-lines in all three generations. Only the results of the T3 generation are presented in Table 1 for statistical reason. When the WT and three null-lines first flowered at an age of 40-d, AtPAP2 OE lines had already produced more branches (Figure 1).

**Table 1:** Flowering time of T3 *Camelina*

	OE6	OE14	OE20	Null-line 6	Null-line 14	Null-line 20	WT
Days to flowering	43 ± 2^a^	43 ± 2^a^	43 ± 2^a^	54 ± 2^b^	54 ± 2^b^	54 ± 2^b^	51 ± 3^b^

Plants were raised in a growth chamber under a 16 h light 22°C/8 h dark 18°C photoperiod/temperature regimen, and then transferred to a greenhouse after flowering. The results were confirmed in 3 independent experiments. Values are means ± SD (n = 4-6). Different lower case letters indicate significantly different (*p *< 0.05)

### Longer hypocotyls in OE lines

**Figure 2:**
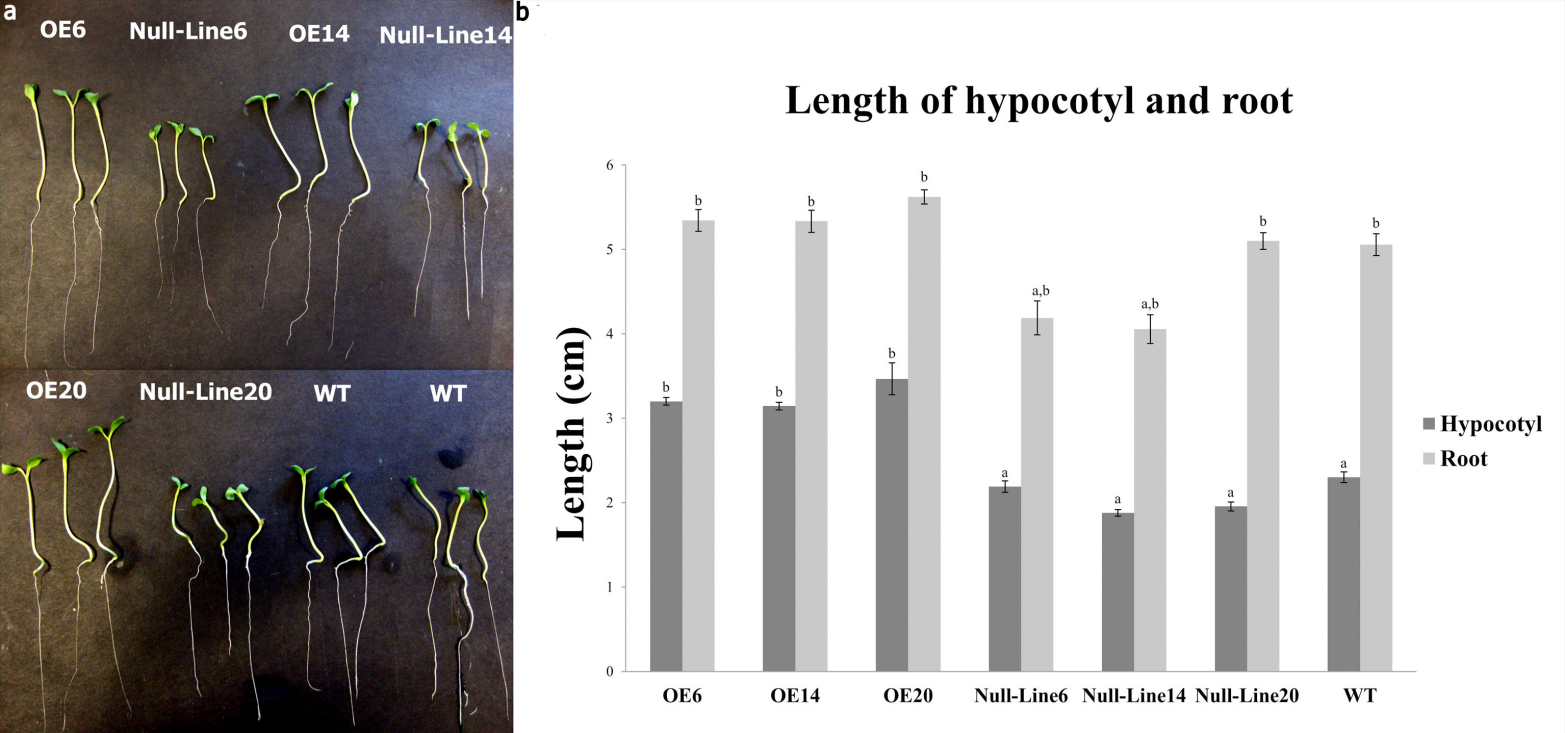
**Five-day old transgenic seedlings grew longer hypocotyls**. a, Three OE lines grew longer hypocotyls than WT and null-line plants. b, Lengths of hypocotyls and roots. Transgenic lines had longer hypocotyls, but there was no significant variation in root length among lines. Values are means ± SD (n = 9 - 14). Different lower case letters indicate significantly different means (*p *< 0.05).

**Figure 3:**
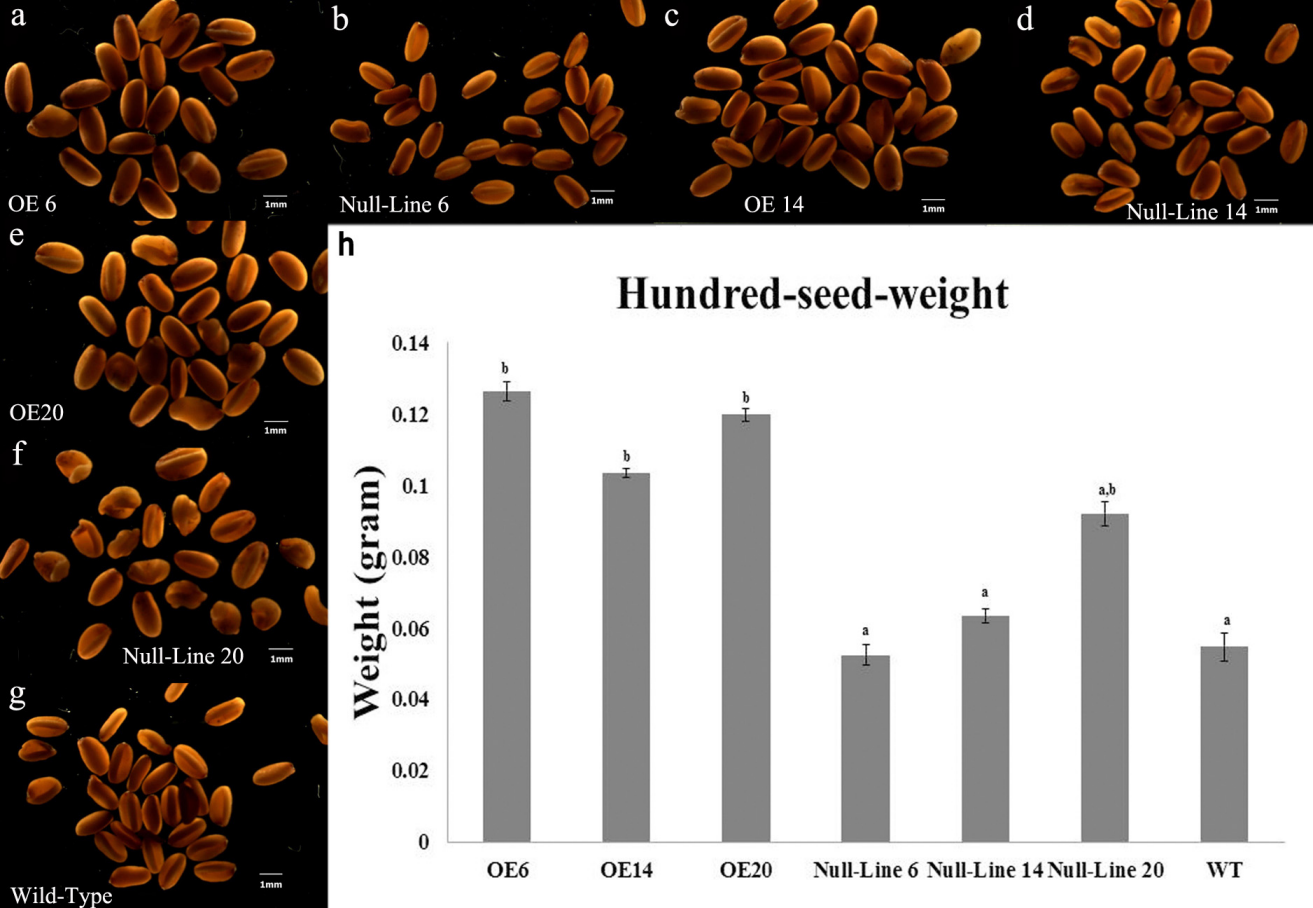
**Seed sizes and hundred-seed-weights of T3 homozygous lines**. Morphology of seeds harvested from OE6 (a), null-line 6 (b), OE14 (c), null-line 14 (d), OE20 (e), null-line 20 (f) and WT (g) plants. All three OE lines produced larger seed size than null-lines and WT plants. (h) The seeds from all three overexpressor lines had higher-hundred-seed weights. Values are means ± SD (n = 4). Different lower case letters indicate significantly different means (*p *< 0.05).

Homologous seeds of three OE lines, three null-lines and the WT were germinated on MS plates and the lengths of hypocotyls and roots were measured 5 days after germination. The three OE lines had longer hypocotyls than their respective null-lines and WT, but there was no significant variation in lengths of roots among lines (Figure 2). The longer hypocotyls may have resulted from the larger seed size in overexpressor lines (Figure 3).

### OE lines produced larger seeds and had higher seed yields

Seeds were collected from all lines after full maturity. Seed weights and sizes were measured after drying. The transgenic lines produced larger seeds (Figure 3a-g) and the hundred-seed-weights from OE plants were 50 ~ 110% higher than those of WT and null-lines (Figure 3h), which may be attributable to an elevated supply of photoassimilates in OE plants. Larger seeds may provide more carbon for seed germination and seedling growth, with correspondingly longer hypocotyls (Figure 2). Generally, total seed yield of OE lines was significantly higher than WT or null-line yields (Table 2), which we attribute to the higher photosynthetic rate in the overexpressors (Table 3). There was no significant difference in germination rates among WT, null-lines and the OE lines (data not shown).

**Table 2:** Higher seed yield from transgenic *Camelina *lines

Seed Yield (g/plant)	OE6	OE14	OE20	Null-Line 6	Null-Line14	Null-Line20	WT
Trial I	1.73 ± 0.16^c^	1.49 ± 0.20^c^	1.04 ± 0.24^cb^	0.85 ± 0.23^ab^	0.66 ± 0.14^a^	0.76 ± 0.24^a^	0.89 ± 0.18^a^

Trial II	2.22 ± 0.22^c^	2.24 ± 0.27^c^	2.09 ± 0.14^c^	1.41 ± 0.07^ab^	0.89 ± 0.25^b^	0.74 ± 0.21^b^	1.08 ± 0.16^a^

Plants were grown in a growth chamber under a 16 h light 22°C/8 h dark 18°C temperature/photoperiod regimen and then transferred to a greenhouse after flowering. Values are means ± SD (n = 4-6). Within each row, values marked by different lower case letters are significantly different (*p *< 0.05). Trials I and II were independent experiments carried out in different growing seasons. OE, overexpressed line; WT, wild type.

**Table 3:** Photosynthesis rates of mature leaves (T3)

Photon flux (μmol m^-2 ^s^-1^)	WT	OE6	OE14	OE20	Null-line 6	Null-line 14	Null-line 20
200	7.68 ± 0.72^abc^	9.12 ± 0.45^b^	8.34 ± 1.0^ab^	8.09 ± 0.65^abc^	8.27 ± 0.45^ab^	7.16 ± 1.07^ac^	6.56 ± 0.26^c^

400	11.06 ± 1.34^a^	14.73 ± 0.62^b^	12.54 ± 1.41^ab^	12.45 ± 0.90^ab^	11.30 ± 0.62^a^	10.29 ± 1.58^ac^	8.82 ± 0.70^c^

600	12.93 ± 1.42^ab^	16.27 ± 0.67^b^	15.13 ± 1.20^b^	15.12 ± 0.40^ab^	12.93 ± 0.67^ab^	11.81 ± 1.78^ac^	9.67 ± 0.97^c^

800	14.13 ± 1.55^a^	17.89 ± 0.61^b^	17.15 ± 0.96^b^	16.50 ± 0.32^b^	13.96 ± 0.61^a^	12.7 ± 1.70^ac^	10.39 ± 1.04^c^

1000	15.02 ± 1.50^a^	19.02 ± 0.69^b^	18.35 ± 1.24^b^	17.64 ± 0.52^b^	14.69 ± 0.69^a^	13.51 ± 1.02^ac^	10.91 ± 0.95^c^

### Higher photosynthetic rates and stomatal conductances in OE lines

Photosynthetic activity and stomatal conductances were measured with a Licor LI-6400XT Portable Photosynthesis System. Third mature leaves below the shoot tips of all plants (35- 37-d old) grown in the growth chamber were used for measurements. The OE lines had higher rates of photosynthesis than WT plants and null-lines at photon flux densities over 200 μmol m^-2 ^s^-1 ^(Table 3). In general, photosynthetic activities per unit leaf area were elevated by 10-25% in overexpressors. As the OE lines had both more branches (and thus greater leaf areas) and higher photosynthetic rates per unit leaf area, each of the OE plants was able to fix more CO_2 _than plants of other lines. In photosynthetic leaves, stomatal conductance is coordinated with CO_2 _requirements of the mesophyll [17]. In our test system, transgenic plants had greater stomatal conductances than WT and null-line plants, in congruence with the elevated photosynthetic rates of overexpressors (Table 4).

**Table 4:** Stomatal conductance of mature leaves (T_3_)

Photon flux (μmol m^-2 ^s^-1^)	WT	OE6	OE14	OE20	Null-line 6	Null-line 14	Null-line 20
200	0.16 ± 0.08^a^	0.27 ± 0.07^b^	0.17 ± 0.08^a^	0.18 ± 0.10^a^	0.16 ± 0.03^a^	0.16 ± 0.13^a^	0.13 ± 0.05^a^

400	0.17 ± 0.07^a^	0.28 ± 0.05^b^	0.19 ± 0.07^a^	0.20 ± 0.08^a^	0.18 ± 0.04^a^	0.16 ± 0.09^a^	0.14 ± 0.04^a^

600	0.20 ± 0.06^a^	0.32 ± 0.04^b^	0.22 ± 0.06^a^	0.25 ± 0.07^a^	0.21 ± 0.05^a^	0.18 ± 0.07^a^	0.15 ± 0.03^a^

800	0.23 ± 0.05^a^	0.37 ± 0.04^b^	0.27 ± 0.05^ab^	0.29 ± 0.07^ab^	0.24 ± 0.06^a^	0.20 ± 0.06^a^	0.17 ± 0.03^a^

1000	0.27 ± 0.04^ab^	0.41 ± 0.03^c^	0.33 ± 0.04^b^	0.32 ± 0.08^b^	0.27 ± 0.07^ab^	0.22 ± 0.06^a^	0.19 ± 0.03^a^

Measurement was carried out on leaves (3^rd^-4^th ^leaves below the shoot tips) of 35-37-d old *Camelina*. Photosynthetic rates (μmol CO_2_/m^2^/s) and stomatal conductance (H_2_O/m^2^/s) were measured at different photon fluxes (0-1000 μmol m^-2 ^s^-1^). Within each row, the values marked by different letters (a, b) are significantly different (*p *< 0.05), n = 3 ~ 5.

### Lower sucrose content in leaves of OE lines

Sugars and sugar-derived metabolic signals affect source-sink balances and carbon partitioning in plants [3,18]. Sugars trigger global repression of photosynthetic gene transcription in plants [4]. To determine whether there were changes in sugar level, we used HPLC methodology to analyze sugar content in leaves of 35-day old *Camelina*. While hexose contents of three AtPAP2 overexpressors were not significantly different from those of the WT and three null-lines, the sucrose contents in leaves of all three transgenic lines were 20 ~ 30% lower in the transgenic plants (Table 5). The low levels of sucrose in leaves may have enhanced photosynthesis, growth rate and biomass accretion [19].

**Table 5:** Sugar contents in leaves

	WT	Null-line 6	Null-line 14	Null-line 20	OE6	OE14	OE20
Hexose	1.78 ± 0.17^a^	1.32 ± 0.20^b^	1.54 ± 0.21^b^	1.39 ± 0.30^b^	1.45 ± 0.15^b^	1.10 ± 0.14^bc^	1.47 ± 0.48^b^

Sucrose	9.42 ± 1.36^a^	8.55 ± 0.61^a^	8.23 ± 2.00^a^	8.83 ± 0.80^a^	7.13 ± 1.12^b^	6.00 ± 0.33^b^	6.38 ± 1.42^b^

Third and fourth fully mature leaves below the shoot tips were used for measurement. Sugar contents are expressed in μg sugar/mg DW (dry weight). Values are means ± SD (n = 4). Within each row, the values marked by different lower case letters are significantly different (p < 0.05). OE, overexpressed line; WT, wild type.

### Increased SPS activity in OE lines

Sucrose phosphate synthase (SPS) is a key enzyme in the sucrose synthesis pathway; it catalyzes conversion of frucose-6-phosphate (Fru6P) and UDP-Glucose (UDPG) to sucrose-6-phosphate [20]. Overexpresssion of SPS in *Arabidopsis *[21] and tobacco [22] affects leaf carbohydrate contents and whole plant development. The phenotypes of *Camelina *AtPAP2 OE lines may be attributable to enhanced enzyme activity during sucrose synthesis. SPS activity was elevated in leaves of AtPAP2 OE plants at both optimal V_max _and limiting V_limit _capacities (Table 6). Higher photosynthetic rates (Table 3) and SPS activity in OE lines may have supplied more sucrose for growth.

**Table 6:** Enhanced SPS Enzyme activities in OE lines

	WT	Null-line 6	Null-line 14	Null-line 20	OE6	OE14	OE20
V_max_	13.1 ± 0.6^a^	14.1 ± 1.6^a^	13.2 ± 1.3^a^	12.8 ± 0.4^a^	16.4 ± 2.2^b^	21.3 ± 3.8^b^	16.0 ± 1.1^b^

V_limit_	2.7 ± 0.5^a^	3.4 ± 0.5^a^	2.6 ± 0.3^a^	3.0 ± 0.7^a^	4.8 ± 1.0^b^	5.8 ± 1.4^b^	5.0 ± 0.5^b^

The third and the fourth fully expanded leaves below the shoot tips were used for SPS enzyme activity analysis after photosynthetic measurements. Enzyme activity is in units of μmole sucrose/mg protein/h. Values are means ± SD (n = 4).Within each row, values marked by different lower case letters are significantly different (*p *< 0.05), n = 4. OE, overexpressed line; WT, wild type.

### Unaltered protein expressions of FBP aldolase SnRK1.1 (and its phosphorylation status), nitrate Reductase, and FBPase in OE lines

SnRK1 protein kinase in plants balances cellular energy levels by regulating ATP production and consumption; this enzyme is activated in response to carbon starvation and energy-depleting stress conditions [23]. SnRK1 phosphorylates central enzymes involved in primary metabolism (e.g. SPS, nitrate reductase), leading to their inactivation [3,24]. We analyzed protein expression levels of SnRK1 and its phosphorylation status by western blotting (Additional file 1). There were no differences in SnRK1 protein expression level or its phosphorylation status among the lines, indicating that higher SPS activity in the OE lines had not resulted from the action of SnRK1 on SPS. Western blotting demonstrated that protein expression levels of other metabolic enzymes, including nitrate reductase (NR), FBPase and FBP aldolase, were unaltered in transgenic plants (Additional file 1). Because anti-AtSPS antibodies from Agrisera (Vännäs, Sweden) did not cross-react with *Camelina *SPS, we were unable to determine the levels of SPS protein.

### Life cycle analysis (LCA) of *Camelina*-based biodiesel (green diesel) produced from seeds of genetically unaltered and transgenic lines

SimaPro LCA software is commonly used to construct LCAs of biodiesel produced from different feedstocks. To build a complete life cycle flow tree for the analysis, it is necessary to first build individual life cycle processes in SimaPro. These are then combined into an assembly, and finally into the life cycle as a whole. The functional unit, as well as the point of junction, is one megajoule (MJ) of energy output [25]. Additional file 2 depicts the life cycle flow tree of *Camelina*-derived fuel from Group 1, namely the group of *Camelina *seed that were genetically unaltered.

**Figure 4:**
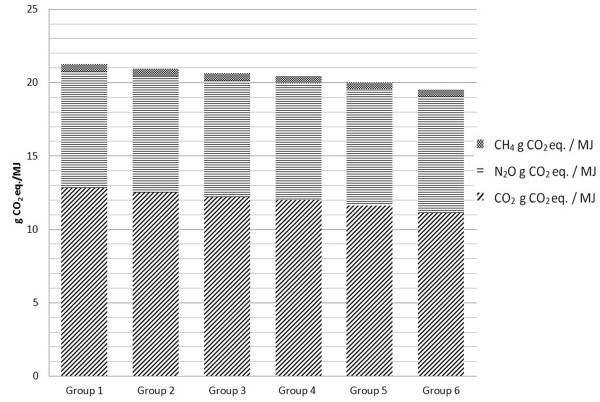
**Global warming potential for different *Camelina *cultivation scenarios**. Impact assessments were calculated with IPCC 2007 Global Warming Potential (GWP) 100a v1.02 software; all GHG emissions are presented in terms of CO_2 _equivalent per MJ energy in oil output for the six *Camelina *cultivation scenarios (see Materials and Methods: Fuel Pathways for an explanation of groupings within the cultivation scenarios). GHG (greenhouse gas) emissions include CO_2_, CH_4_, N_2_O and the minor contributions from solvents and refrigerants in CO_2 _equivalents.

The outputs of genetically modified *Camelina *seed yields ha^-1 ^were assumed to increase by 10% (Group 2), 20% (Group 3), 30% (Group 4), 50% (Group 5) and 100% (Group 6) above yields obtained from farming of genetically unaltered *Camelina *(Additional file 3). The two impact assessment methods selected for this study were IPCC 2007 Global Warming Potential (GWP) 100a v1.02 and Cumulative Energy Demand (CED). The output for IPCC 2007 GWP is in CO_2 _equivalents for all of the greenhouse gas (GHG) emissions, including CO_2_, CH_4_, N_2_O and minor contributions from solvents and refrigerants. The second impact assessment method calculates CED for different categories, including non-renewable fossil energy or renewable biomass energy, as MJ-equivalent energy input per MJ energy output. Figure 4 shows GWP, and presents emissions of GHG in terms of CO_2 _equivalents for the six *Camelina *cultivation scenarios. The declining GWP from Group 1 through Group 6 demonstrates that emissions of CO_2 _equivalent per MJ energy of oil output decreased with increasing seed yields. Table 7 compares CED across the six scenarios. There is a similar slightly decreasing trend from Group 1 to Group 6, indicating a moderate decrease in energy demand with increasing seed yields. As mentioned, seed yields of OE6 and OE14 lines were improved by about 100% in greenhouse conditions; these two lines would thus fall into Group 6 of the simulation. The LCA results for Group 6 indicate 8.2% and 2.3% reductions in GWP and CED, respectively, in comparison with Group 1. Thus, transgenic lines would be more environmentally friendly if yield improvement is reproducible under field conditions.

**Table 7:** Cumulative Energy Demands

Impact Category-	Group 1	Group 2	Group 3	Group 4	Group 5	Group 6
Non-renewable, fossil	0.224	0.219	0.215	0.212	0.207	0.198

Renewable, biomass	1.04	1.04	1.04	1.04	1.04	1.04

*Others	0.012	0.012	0.012	0.012	0.011	0.011

Total	1.28	1.27	1.27	1.26	1.26	1.25

*Others include non-renewable biomass and renewable energy sources, such as solar, wind and water, that may be used in the process. The Group 1 column lists the energy demands for cultivation of wild-type *Camelina *seed; columns under Groups 2, 3, 4, 5 and 6 list demands for cultivating genetically modified plants with various levels of yield improvement (10%, 20%, 30%, 50% and 100% mass increases per unit area, respectively). Cells contain ratios of MJ (input)/MJ (output).

## Discussion

Sugar metabolism plays important roles in vegetative growth and reproductive development of plants. Sugars affect expression of genes that encode carbon metabolic enzymes [3,26]. Developmental processes, such as flowering [27], fruiting [28] and the sink to source transition [29], may be improved by overexpression of SPS. In the present study, overexpression of *Arabidopsis *AtPAP2 in transgenic *Camelina *boosted activity of SPS in leaves, similar to the effects in transgenic *Arabidopsis *[5]. Although SPS activity in leaves was elevated, there was a surprising decrease in leaf sugar contents in the OE lines, contrary to results for transgenic *Arabidopsis *[5]. The sucrose levels in leaves of 35-d old *Camelina *were reduced by 20%, suggesting an elevated sucrose export rate in the transgenic lines. The rate of sucrose synthesis under the control of SPS is correlated with the rate of export from leaves [30] and also with the rate of photosynthesis [31]. Higher photosynthetic rates lead to increases in leaf sucrose, while the rate of sucrose export from leaves determines the abundance of sucrose at the source [32]; low leaf sucrose content in overexpressed *Camelina *lines may have resulted from highly efficient transport of sucrose. High sucrose content in leaves suppresses photosynthesis. Reduced sucrose content in source leaves may alleviate feedback inhibition of photosynthesis and SPS activity caused by high sugar concentrations. Overall, this would enhance carbon fixation and SPS activity. As the major transport carbohydrate in plants, sucrose links carbon assimilation in source tissues to carbon utilization in sink tissues [18,33]. Elevated photosynthesis produces more carbon skeletons, while enhanced efficiencies of sucrose synthesis and transport increase sugar supply to sinks, resulting in earlier flowering, faster growth and improved seed yield in transgenic lines.

LCA analysis indicated that improved seed yield of transgenic *Camelina sativa *lines may prove useful in the production of resource-economical green biodiesel with reduced Cumulative Energy Demand and Global Warming Potential. Compared to petroleum jet fuel, the GHG emission of *Camelina*-based biofuels was reduced by 68-75% (> 67%) at the current yield level from genetically unaltered plants. This could be further improved to 75-80% by increasing seed yield per unit area. While the transgenic *Camelina sativa *produced a higher seed yield under controlled environmental conditions, whether the transgenic lines could perform well under field conditions needs further experiments. Growth density, availability of water, competition of nutrition, pest damages, may adversely affect the growth and yield of the transgenic lines. Nonetheless, with expected future gains in seed yield, *Camelina *production in USA has the potential to provide three billion liters per year of high quality, environmentally-friendly jet fuel [25]. Commercial use of *Camelina*-based jet fuel is expected in 2012.

## Conclusion

Overexpression of AtPAP2 promoted *Camelina *growth through effects on carbon assimilation and distribution. More importantly, improved seed yield could enhance the value of *Camelina *as a low-input crop for biofuel production and for various industrial and agricultural applications.

## Methods

### Plant materials, growth conditions, and vector

*Camelina *seeds were thrice surface-sterilized with 20% (v/v) bleach for 15 min; the seeds were then washed with sterile distilled water thrice before plating onto MS [34] medium supplemented with 2% (w/v) sucrose for 5 d. The seedlings were then transferred into 15-cm-diameter pots filled with sterilized soil. *Camelina *plants were then grown in a growth chamber (150 μmol photons m^-2 ^s^-1^; 75% RH) under a16 h light 22°C/8 h dark 18°C temperature/photoperiod regimen and watered every week with distilled water. Collected seeds were dried at 37°C in an incubator one week before use. Total RNA was isolated from fresh leaves by using Trizol reagent (Invitrogen, HK.). To generate full-length cDNA for RT-PCR, reverse transcription was performed using M-MLV reverse transcriptase (Promegra, HK). For *Camelina *transformation, full length *AtPAP2 *(At1g13900, TAIR Database) cDNA was amplified with Platinum^® ^*Pfx *(Invitrogen, HK) and subcloned into the plant transformation vector pBa002 with Xhol and Sacl.

### Agrobacterium-mediated transformation

Six-week old WT *Camelina *plants that first started flowering in the growth chamber were used for transformation following methodology of Lu et al. [14]. After first transformation, new flowers in inflorescences were used for second and third transformations to improve the transformation ratio. After the third transformation, new inflorescences were cut to reduce background.

### Generation of *Camelina *overexpression lines and null-lines

Homologous AtPAP2 transgenic lines of *Camelina *were selected on Basta plates (5 g/l Basta, Riedel-deHaen, Germany, 500 g/l carboncillin, 100 seeds/plate); WT *Camelina *was used as the negative control. Resistant plants were transferred to soil for growth to maturity. Their transgenic status was confirmed by genomic PCR and western blotting analyses using anti-AtPAP2 antibodies. Homozygous T_3 _seeds of the transgenic plants were used for further analyses. When there was 3:1 segregation of T_1 _transgenic seeds on Basta plates, null-lines were selected by their shorter hypocotyl lengths on MS plates. Identities of the three null-lines were confirmed by genomic PCR.

### Seed viability, root and hypocotyl length measurements

*Camelina *seeds were surface sterilized with 20% (v/v) bleach for 15-20 min, washed with sterile distilled water and then plated on MS medium (50 seeds/plate). The experiment was carried out in a growth room (~100 μmol photons/m^2^/s) under a 16 h light (22°C)/8 h dark (18°C) temperature/photoperiod regimen. The lengths of roots and hypocotyls were measured 5 d after germination. Viability tests on *Camelina *seeds were performed in 10-cm diameter Petri plates each containing 20 ml of MS medium supplemented with 2% (w/v) sucrose; results were recorded after 5-6 d.

### Extraction of plant genomic DNA and genomic PCR

Genomic DNA was extracted from young leaves using a CTAB-based method [35]. The 25 μl reaction mixture contained ~200 ng of genomic DNA, 0.5 μl of each primer (15 mmol/l), 0.5 μl of dNTP mix (10 mmol/l each), 5 μl of green GoTaq reaction buffer and 0.25 μl of GoTaq DNA polymerase (Promega, HK). Cycling began with one cycle at 95°C for 2 min, followed by 30 cycles at 95°C for 30 s, 50°C for 30 s, 72°C for 150 s and a final extension at 72°C for 10 min. PCR products were sequenced using the primers AtPAP2-f (5'-TGCACTCGAGATGATCGTTAATTTCTCTTTC-3') and AtPAP2-r (5'-GTACGAGCTCTTATGTCTCCTCGTTCTTG-3'), and analyzed by electrophoresis in 1.0% (v/v) agarose gels.

### Western blot analysis

*Camelina *leaves were finely ground in 1.5-ml Eppendorf tubes each containing 200 μl of ice-cooled extraction buffer (50 mMTris-HCl, pH 7.4 containing 150 mMNaCl, 1 mM EDTA, and 0.2 mM PMSF) and incubated on ice for 30 min with occasional mixing. The protein extract was separated by centrifugation at 4°C for 30 min at 10,000 × *g*. The supernatants were transferred to new 1.5-ml Eppendorf tubes and protein concentrations were determined with a Bio-Rad (Hercules, CA, USA) Protein Assay Kit. Proteins separated on 8% (v/v) SDS-PAGE were transferred to Hybond C-Extra membranes (GE Healthcare, HK) (400 mA, 1 h). Membranes were blocked with 5% (w/v) non-fat milk in TBST buffer (20 mMTris-HCl, pH 7.6, 137 mMNaCl, 0.1% (v/v) Tween 20) for about 2 h and probed with specific antiserum for 3 h at room temperature or overnight at 4°C. The membrane was rinsed with TBST, and HRP-labeled secondary antibody (1:10,000) in TBST was added for one more hour. The proteins were visualized with the Enhanced Chemiluminescence method (GE Healthcare, HK).

### Measurement of photosynthetic rates and stomatal conductances

Photosynthetic rates and stomatal conductances were measured using a portable photosynthesis system (LI-COR, LI-6400, Nebraska, USA) in the morning (08:30-12:30) under a fixed blue-red light-emitting diode (LED) light source. Ten measurements were made on each fully expanded leaf collected from the shoot tips of 35-37-d old plants; at least 3 plants from each line were used for measurements. The leaf area of the standard broadleaf chamber was 6 cm^2 ^(2 × 3 cm) and a fixed light source was provided by an array of red and blue LEDs. *P *vs. *I *curves were plotted using the instrument's auto-program function. Measurements were taken at light intensities of 200, 400, 600, 800 and 1000 μmol photons/m^2^/s.

Photosynthetic rate (A) was calculated as A = F(Cr-Cs)/100S-CsE, where A = net assimilation rate (μmol CO_2_/m^2^/s^1^), F = molar flow rate of air entering the leaf chamber (μmol/s), Cr = mole fraction of CO_2 _in the reference IRGA (infra-red gas analyzer) (μmol CO_2_/mol air), Cs = mol fraction of CO_2 _in the sample IRGA (μmol CO_2_/mol air), S = leaf area (cm^2^), and E = transpiration rate (mol H_2_O/m^2^/s).

Stomatal conductance is the rate at which water evaporates from stomata; it is directly related to the relative size of the stomatal aperture. Conductance is calculated from gsw = 1/(1/gtw-Kf/gbw), where gsw = stomatal conductance to water vapor (mol H_2_O/m^2^/s), gtw = total conductance to water vapor (mol H_2_O/m^2^/s), Kf = stomatal ratio, and gbw = boundary layer conductance to water vapor (μmol H_2_O/m^2^/s).

### HPLC analysis of sugar content

For measurement of sucrose and hexose of plant tissues, 0.1 g aliquots of freeze-dried tissue powder were resuspended in 1 ml of 70% (v/v) ethanol, incubated at 70°C for 90 min and centrifuged at 13,000 × *g *for 10 min. After passing through a 0.22 mm filter, a 10 μl sample was injected into a CarboPac PA 1 column (4 × 250 mm) connected to a Dionex (Sunnyvale, CA, USA) LC 20 Chromatography system and sugar contents were analyzed by high performance anion exchange chromatography with pulsed amperometric detection [36]. Standard curves were prepared with 0-0.1 mg/ml sucrose, fructose and glucose solutions in 70% (v/v) ethanol.

### SPS activity assay

SPS activity was assayed by the anthrone test [22]. Samples were incubated for 20 min at 25°C in 50 μl of pre-balanced buffer (50 mM HEPES-KOH pH 7.5, 20 mMKCl, and 4 mM MgCl_2_) containing (a) (for V_max _assay) 12 mM UDPG and 10 mM Fru6P (in a 1:4 ratio with glucose-6-phosphate (Glc6P), and (b) (for V_limiting _assay) 4 mM UDPG and 2 mM Fru6P (in a 1:4 ratio with Glc6P) and 5 mM KH_2_PO_4_.

### Implementation of SimaPro software for life cycle analysis of green diesel from unaltered *Camelina *and its genetically modified counterparts

SimaPro was used to construct a LCA (life cycle analysis) of bio-diesel from wild type and genetically modified plants with different seed yields. The general processes for initiating a LCA study in SimaPro include: i. Determining the goal and scope of the specific study, which define the LCA system boundary. ii. Building of the life cycle flow tree, which specifies each stage involved in the life cycle. All environmental inflow and outflow inventory data are collected at this stage to build the life cycle processes; relevant life cycle assembly is then undertaken, followed by final construction of life cycles. Waste scenarios may be added into the life cycle flow tree, if available. iii. Choosing appropriate life cycle impact assessment methodology to calculate prospective results. iv. Interpreting results.

### Fuel pathways

Life cycle analysis was conducted for biofuel derived from unmodified *Camelina *seeds (Group 1) and for biofuels derived from genetically modified *Camelina *with enhanced seed yields. We assumed that all input parameters were fixed and that the outputs of genetically modified *Camelina *seed yield increased by 10% (Group 2), 20% (Group 3), 30% (Group 4), 50% (Group 5) and 100% (Group 6) over output from the unmodified *Camelina *farming process. The whole life cycle was divided into several stages, including *Camelina *farming, seed oil production, refined oil production and end use oil output, which also set the system boundary defined for the study. The life cycle stages were used to organize data for software calculations.

### Functional unit and life cycle inventory

The function unit in this study was one MJ of energy content in the fuel output. The choice was appropriate since energy content is the fundamental measurement for all environmental and energy flows in the life cycle analysis. The life cycle inventory for every stage came from the public *Camelina *database for *Camelina *in Montana, USA and the database in SimaPro. The input data for *Camelina *farming, seed oil extraction, refined oil production and transportation are shown in Additional file 3, 4 and 5.

As shown in Additional file 3 Group 1 represented the original *Camelina *seed yield, and Groups 2, 3, 4, 5 and 6 represented the yield increases in genetically modified plants, with seed yields increasing by 10%, 20%, 30%, 50% and 100% per unit area, respectively. The cumulative inputs of potassium (as K_2_O), thomas meal fertilizer (as P_2_O_5_) and urea-N were fixed for the one-hectare farming input. The quantity of fossil fuel diesel used remained unchanged on a unit area basis but changed with changing *Camelina *seed output. As for GHG emissions, methane and dinitrogen monoxide emissions per kilogram of seed output remained unchanged. Carbon dioxide emissiona from diesel changed linearly with changing fossil diesel consumption, but at the same time remained fixed per unit area farmland output.

### Growth phenotypes measurement, seed morphology, flowering time, and data analysis

Duration of time to first bud opening in the primary inflorescence was used as a measure of flowering time in *Camelina*. Flowering times were recorded in the T1, T2 and T3 generations (n = 4-6). Seed morphology of *Camelina *was observed microscopically. In Tables 1, 2, 3, 4, 5, 6 statistical differences (P < 0.05) in the same row for each line were based on one-way ANOVA analysis followed by Tukey's Honestly Significant Differences (HSD) test using statistical program SPSS 18.

## Abbreviations

AtPAP2: *Arabidopsis *purple acid phosphatase 2; CED: Cumulative Energy Demand; FBP: Fructose 1,6-bisphosphate; FBPase: Tfructose 1,6-bisphosphatase; Fru6P: Frucose-6-phosphate; GHG: Greenhouse gas; GWP: Global Warming Potential; IRGA: Infra-red gas analyzer; LCA: Life cycle analysis; LED: Light-emitting diode; MJ: Megajoule; NR: Nitrate reductase; SPS: Sucrose phosphate synthase; PAP: Purple acid phosphatase; OE: Overexpression; UDPG: UDP-Glucose; WT: Wild-type.

## Competing interests

The authors declare that they have no competing interests.

## Authors' contributions

YZ designed the study, performed the experiments, analyzed the results and wrote the manuscript. BLL designed the study, analyzed the results and revised the manuscript. LY and DL performed life cycle analysis, participated in manuscript preparation and commentary. FS participated in vector preparation. All authors participated in correction of the manuscript, and approved the final version.

## Supplementary Material

Additional file 1 **Western blot analysis of NR, FBP aldolase, cFBPase, SnRK1 and its phosphorylation status**. Click here for file

Additional file 2 **Life cycle flow tree for Group 1 camelina green diesel**. Click here for file

Additional file 3 **Cultivation inputs and outputs for camelina farming**. Click here for file

Additional file 4 **Processing inputs for camelina seed oil production**. Click here for file

Additional file 5 **Processing inputs for camelina refined oil production**. Click here for file
