# Supplementary material for: Over-expression of AtPAP2 in Camelina sativa leads to faster plant growth and higher seed yield
Source: Biotechnol Biofuels. 2012 Apr 2;5:19. doi: 10.1186/1754-6834-5-19 (PMC3361479; doi:10.1186/1754-6834-5-19)
Supplement: Additional file 1 — Western blot analysis of NR, FBP aldolase, cFBPase, SnRK1 and its phosphorylation status. [file 1754-6834-5-19-S1.DOC]

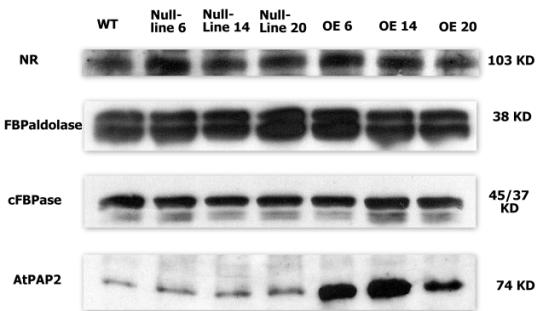


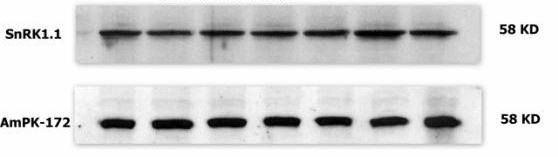


**Additional file 1. Western blot analyses of NR, FBP aldolase, cFBPase and SnRK1 (and its phosphorylation status).** Total soluble proteins were extracted from 30-day-old plants in the middle of day. cFBPase, cytosolic fructose-1,6-bisphosphatase; anti-cFBPase, Agrisera AS04043, Sweden; anti-FBP aldolase, Agrisera AS08294 Sweden; NR, nitrate reductase; anti-NR, Agrisera AS08310 Sweden; anti-AtPAP2, AtPAP2 specific antibody; SnRK, Snf1-related kinase 1; and anti-AtSnRK1.1 were provided by Sun Feng. anti-AMPK172, AMPK-T172 phosphorylated antiserum (Cell Signaling Inc., Beverly, MA)**;** cFBPase was used as an equal protein loading control.
