# Supplementary material for: Over-expression of AtPAP2 in Camelina sativa leads to faster plant growth and higher seed yield
Source: Biotechnol Biofuels. 2012 Apr 2;5:19. doi: 10.1186/1754-6834-5-19 (PMC3361479; doi:10.1186/1754-6834-5-19)
Supplement: Additional file 2 — Life cycle flow tree for Group 1 camelina green diesel. [file 1754-6834-5-19-S2.DOC]

**
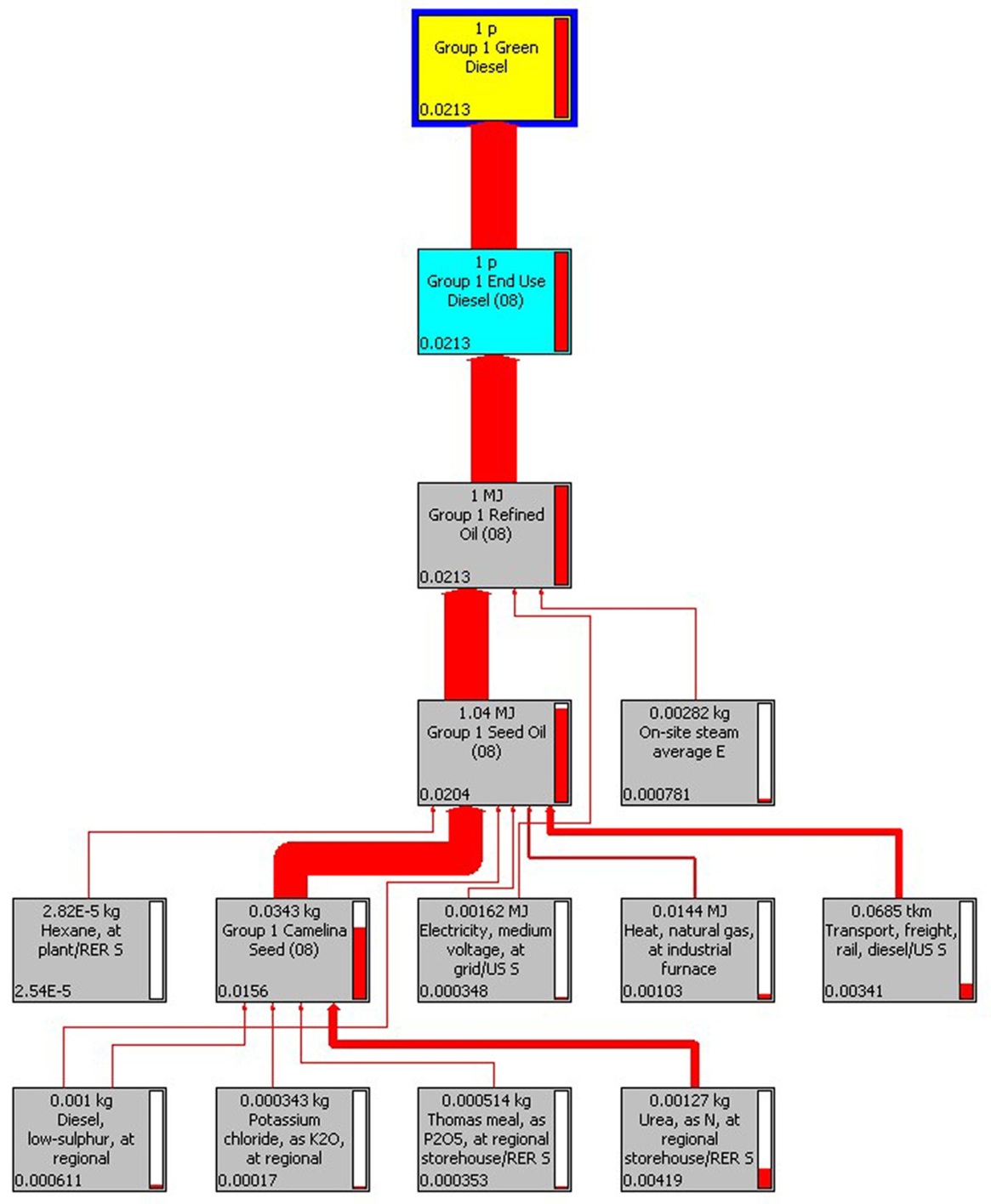
**

**Additional file 2. Life cycle flow tree for Group 1 Camelina green diesel.** Each grey box indicates a process; the turquoise blue box represents a (sub) assembly. The small red bar charts (or thermometers) indicate the environmental load generated in each process and its upstream processes. The figure indicates all the environmental inflow and outflow inventory data collected and calculated in the whole life cycle. To build the flow tree, individual life cycle processes were specified in SimaPro first, and then the processes were combined into the relevant assembly, and finally the life cycle was composed as a whole. The functional unit, which acts as the point of junction to build the flow tree, is 1 megajoule (MJ) of energy output [24].
