# Supplementary material for: Over-expression of AtPAP2 in Camelina sativa leads to faster plant growth and higher seed yield
Source: Biotechnol Biofuels. 2012 Apr 2;5:19. doi: 10.1186/1754-6834-5-19 (PMC3361479; doi:10.1186/1754-6834-5-19)
Supplement: Additional file 3 — Cultivation inputs and outputs for camelina farming. [file 1754-6834-5-19-S3.DOC]

**Additional file 3. Cultivation inputs and outputs for Camelina farming.**

| Cultivation Input/ output (per kg seed) | | Group 1 | Group 2 | Group 3 | Group 4 | Group 5 | Group 6 |
| --- | --- | --- | --- | --- | --- | --- | --- |
| Known Inputs from biomass | Energy content of seed (MJ) | 30.4 | 30.4 | 30.4 | 30.4 | 30.4 | 30.4 |
| Known Inputs from technosphere (materials/ fuels) | Potassium chloride, as K_2_O (kg) | 0.01 | 0.00909 | 0.00833 | 0.00769 | 0.00667 | 0.005 |
|  | Thomas meal, as P_2_O_5_ (kg) | 0.015 | 0.0136 | 0.0125 | 0.0115 | 0.01 | 0.0075 |
|  | Urea, as N (kg) | 0.037 | 0.037 | 0.037 | 0.037 | 0.037 | 0.037 |
|  | Diesel, low-sulfur (kg) | 0.0238 | 0.0216 | 0.0198 | 0.0183 | 0.0159 | 0.0119 |
| Emission to Air | Methane (g) | 0.0024 | 0.0024 | 0.0024 | 0.0024 | 0.0024 | 0.0024 |
|  | Dinitrogen monoxide (g) | 0.77 | 0.77 | 0.77 | 0.77 | 0.77 | 0.77 |
|  | Carbon dioxide (diesel) (kg) | 0.0738 | 0.0671 | 0.0615 | 0.0568 | 0.0492 | 0.0369 |
|  | Carbon dioxide (urea) (kg) | 0.0581 | 0.0581 | 0.0581 | 0.0581 | 0.0581 | 0.0581 |

*Group 1, 2, 3, 4, 5 and 6 represent the Farmer 2008 camelina farming data and the genetic modified ones whose yield were increased by 10%, 20%, 30%, 50% and 100% respectively, over the unmodified crop.
