# Supplementary material for: Over-expression of AtPAP2 in Camelina sativa leads to faster plant growth and higher seed yield
Source: Biotechnol Biofuels. 2012 Apr 2;5:19. doi: 10.1186/1754-6834-5-19 (PMC3361479; doi:10.1186/1754-6834-5-19)
Supplement: Additional file 4 — Processing inputs for camelina seed oil production. [file 1754-6834-5-19-S4.DOC]

**Additional file 4. Processing inputs for Camelina seed oil production.**

| *Processing Input from camelina seed to degummed oil (per kg seed)* | Amount |
| --- | --- |
| *Electricity, medium voltage, average U.S. mix* | 0.0083 kWh |
| *Heat, natural gas, at industrial furnace > 100 kwh* | 0.421 MJ |
| *Diesel, low-sulfur, at regional storage (seed transport)* | 0.0064 L, 5.378 g |
| *Hexane (oil extraction solvent)* | 0.00125 L, 0.82425 g |
| *Rail transport of crude camelina oil* | 2 tkm |
