# Supplementary material for: Over-expression of AtPAP2 in Camelina sativa leads to faster plant growth and higher seed yield
Source: Biotechnol Biofuels. 2012 Apr 2;5:19. doi: 10.1186/1754-6834-5-19 (PMC3361479; doi:10.1186/1754-6834-5-19)
Supplement: Additional file 5 — Processing inputs for camelina refined oil production. [file 1754-6834-5-19-S5.DOC]

**Additional file 5. Processing inputs for Camelina refined oil production.**

| Processing Input for degummed oil to refined oil (per MJ) | Amount |
| --- | --- |
| Camelina crude oil per MJ refined oil | 1.0417 MJ |
| Steam | 0.00282 kg |
| Electricity, medium voltage, average U.S. mix | 0.0006 MJ |
